# The association between blood lipid levels and the risk of atrophic gastritis: a cross-sectional study of the Wuwei cohort

**DOI:** 10.3389/fpubh.2026.1878786

**Published:** 2026-06-30

**Authors:** Bowen Li, Jie Gao, Ya Zheng, Linzhi Lu, Rui Ji, Qinghong Guo, Zhaofeng Chen, Yuping Wang, Hong Lu, Qian Ren, Yongning Zhou

**Affiliations:** 1 The First School of Clinical Medicine, Lanzhou University, Lanzhou, China; 2 Department of Pediatrics, The First Hospital of Lanzhou University, Lanzhou, China; 3 Department of Gastroenterology, the First Hospital of Lanzhou University, Lanzhou, China; 4 Clinical Research Center for Gastrointestinal Diseases of Gansu Province, Lanzhou University, Lanzhou, China; 5 Gansu Wuwei Tumor Hospital, Wuwei, Gansu, China

**Keywords:** atrophic gastritis, blood lipid, cholesterol, cross-sectional study, *Helicobacter pylori*, LDL cholesterol

## Abstract

**Background:**

Aberrant lipid profiles have been associated with several chronic diseases; however, their relationship with atrophic gastritis remains unclear. This study aimed to examine the association between serum lipid levels and the prevalence of chronic atrophic gastritis among participants from the Wuwei cohort.

**Methods:**

We conducted a cross-sectional analysis of 5,194 participants from the Wuwei cohort, including individuals with pathologically confirmed chronic atrophic gastritis and individuals with normal gastric mucosa. Serum lipid parameters, including total cholesterol, triglycerides, high-density lipoprotein cholesterol, and low-density lipoprotein cholesterol, were assessed. Odds ratios and 95% confidence intervals for prevalent chronic atrophic gastritis were estimated using binary logistic regression models.

**Results:**

In Model 1, elevated total cholesterol was identified as a risk factor for the development of chronic atrophic gastritis (CAG) with an odds ratio (OR) of 1.07 (95% CI = 1.01–1.14). Similarly, elevated LDL was associated with an increased risk of atrophic gastritis, with an OR of 1.20 (95% CI = 1.10–1.32) compared to the group without CAG. These associations remained significant in Model 2, with odds ratios of 1.10 (95% CI = 1.03–1.17) for total cholesterol and 1.21 (95% CI = 1.10–1.34) for LDL.

**Conclusion:**

In this cross-sectional study, higher total cholesterol and LDL cholesterol levels were associated with greater odds of prevalent chronic atrophic gastritis.

## Introduction

Atrophic gastritis (AG) is a precancerous condition characterized by the replacement of normal gastric glandular structures with connective tissue (nonmetaplastic atrophy) or a different, non-native epithelium (metaplastic atrophy) in the presence of chronic inflammation (1, 2). AG is part of a series of precancerous lesions within the gastric mucosa, occurring prior to the emergence of the intestinal subtype of gastric cancer. The typical progression involves non-atrophic gastritis progressing to chronic atrophic gastritis (CAG), followed by intestinal metaplasia, abnormal hyperplasia, and eventually adenocarcinoma (3). Understanding the occurrence of gastric cancer and identifying its risk factors requires the study of precursor lesions such as atrophic gastritis and their associated risk factors.

In recent years, abnormal lipid metabolism has become increasingly common alongside changes in lifestyle and dietary patterns. Dyslipidemia has been linked to a range of gastrointestinal and metabolic disorders, but evidence regarding its relationship with atrophic gastritis remains limited and inconsistent. Because atrophic gastritis is an important precancerous gastric lesion, identifying factors associated with its presence may help improve risk stratification and guide future etiological research.

Therefore, using data from the Wuwei cohort, we conducted a cross-sectional study to examine the association between serum lipid profiles and the prevalence of chronic atrophic gastritis. Given the cross-sectional nature of the study, our analyses were intended to assess associations rather than establish temporal or causal relationships.

## Materials and methods

### Study population

Among the 21,345 participants who underwent gastroscopy and pathological assessment in the Wuwei cohort, 1,750 were diagnosed with normal gastric mucosa, 3,444 with chronic atrophic gastritis CAG, 1,788 with intestinal metaplasia, 2,283 with low-grade intraepithelial neoplasia, and 244 with gastric cancer. Because the objective of the present study was to evaluate the association between serum lipid profiles and prevalent CAG, we restricted the analytical sample to participants with either normal gastric mucosa or pathologically confirmed CAG. Participants with intestinal metaplasia, low-grade intraepithelial neoplasia, gastric cancer, or other gastric mucosal diagnoses were excluded to avoid heterogeneity across different stages of gastric mucosal lesions. All participants with normal gastric mucosa and all participants with CAG who met the inclusion criteria were included in the final analysis.

All participants provided written informed consent and underwent comprehensive physical and epidemiological examinations before enrollment. The study was approved by the Ethics Committee of the First Hospital of Lanzhou University approval number: LDYYLL2012001 and was conducted in accordance with the Declaration of Helsinki.

### Exposure and outcome assessment

In this study, covariates considered included sociodemographic characteristics (age, gender, education, occupation, marital status), smoking and alcohol consumption habits, dietary habits (including consumption of hot and fast food), body mass index (BMI), *Helicobacter pylori* infection status, and medical history (gastritis, peptic ulcer). Participants were classified as smokers if they smoked at least one cigarette per day or had smoked within the past 6 months. Alcohol consumers were defined as individuals who consumed 1,000 grams of beer, 150 grams of wine, or spirits at least once per week in the past year. Participants were classified as consuming hot food if they reported a habit of doing so. Those who consumed a bowl of pasta in 8 min or less were considered to have a fast eating habit. Trained staff measured participants’ height and weight, and BMI was calculated as weight in kilograms divided by the square of height in meters. BMI was classified based on Chinese cut-off points: BMI < 23 kg/m^2^ for underweight, 23 kg/m^2^ ≤ BMI 25 kg/m^2^ for normal weight, 25 kg/m^2^ ≤ BMI < 28.0 kg/m^2^ for overweight, and BMI ≥ 28.0 kg/m^2^ for obesity. The 14C-Urea breath test was employed to detect active *H. pylori* infection. Participants ingested a test capsule containing urea labeled with radioactive carbon 14, either on an empty stomach or 2 h after eating. An *H. pylori* detector (Shenzhen Zhonghe Headway BIO-SCI & TECH, China) was utilized to detect the presence of 14C-labeled urea. Participants were classified as having hypertension if they were taking antihypertensive medications, self-reported a history of hypertension, and/or if their systolic pressure was ≥140 mmHg or diastolic pressure was ≥ 90 mmHg at baseline.

### Statistical analysis

The data were analyzed using SPSS 26.0 for Windows SPSS Inc., Chicago, IL, USA. Baseline characteristics were summarized as proportions for categorical variables and as means ± standard deviations or medians with interquartile ranges for continuous variables, as appropriate. Serum lipid concentrations, including TC, TG, HDL-C, and LDL-C, were analyzed on their original scale in mmol/L and were not log-transformed in the primary analyses. When lipid variables were modeled as continuous variables, ORs and 95% CIs were reported per 1 mmol/L increase in the corresponding lipid parameter. The associations between serum lipid levels and prevalent CAG were evaluated using binary logistic regression models and quartile-based analyses. Restricted cubic spline analyses were performed to assess potential nonlinear associations between serum lipid levels and prevalent CAG. All participants with valid serum lipid measurements were included in the primary analyses, and no observations were excluded solely because of high lipid values. Sensitivity analyses were conducted using winsorized lipid values at the 1st and 99th percentiles to assess the influence of extreme values.

## Results

### Characteristics of participants

**Figure 1:**
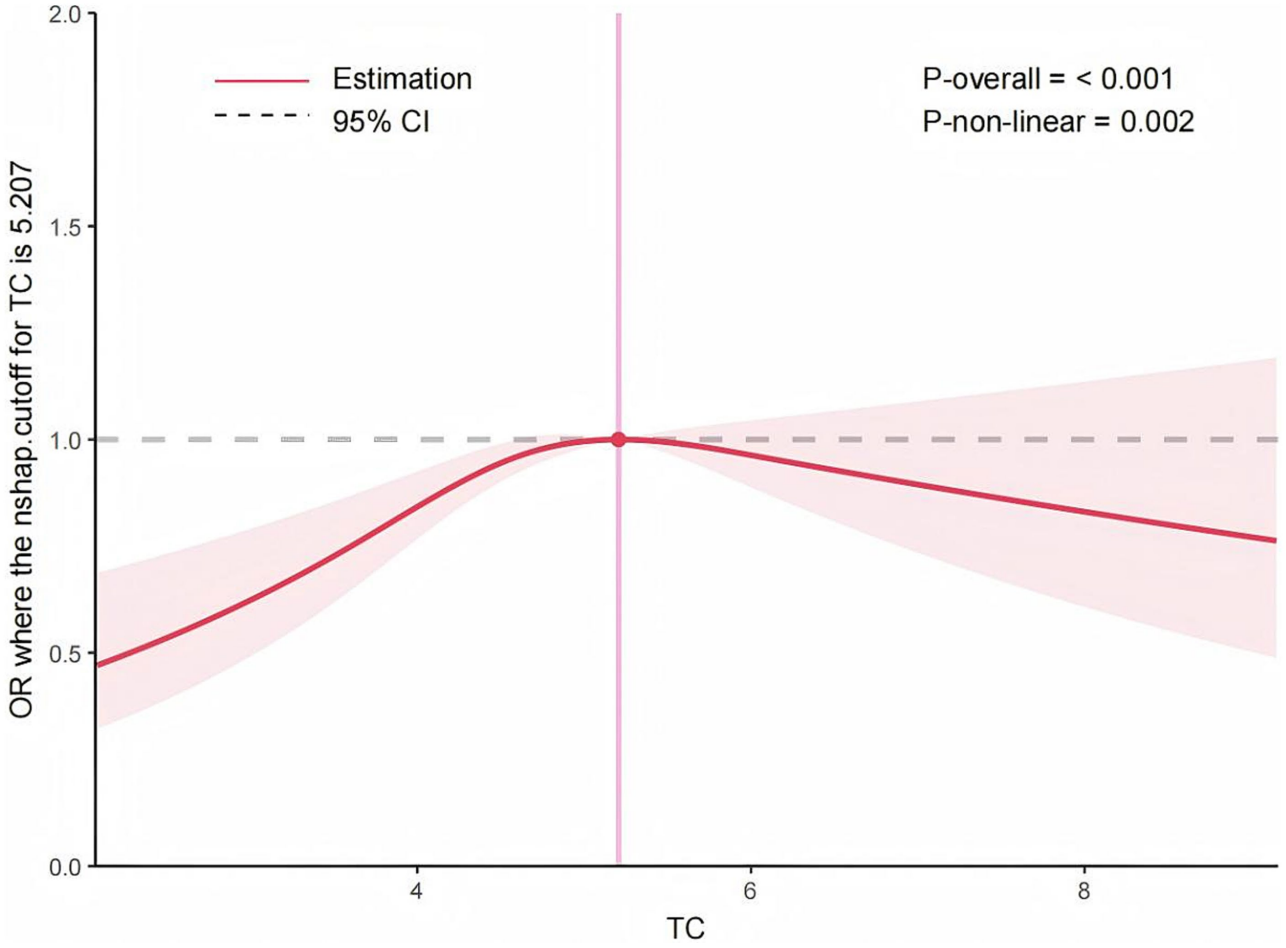
Flow chart of the study population.

The analytical sample included 5,194 participants, of whom 3,444 had chronic atrophic gastritis and 1,750 had normal gastric mucosa. Table 1 shows the basic clinical characteristics of the patients. Patients in the atrophic gastritis group were older compared to the non-atrophic gastritis population and most of them were farmers and low-income earners. Compared to the No CAG group, patients in the CAG group were more likely to be male, more likely to smoke and drink alcohol, more likely to be infected with *Helicobacter pylori*, and more likely to drink alcohol and eat hot food. Total cholesterol and LDL-C levels were higher in the CAG group than in the No-CAG group. (*p* < 0.05) (see Figure 1).

**Table 1:** Baseline characteristics of the study subjects.

Variables	No-CAG	CAG	*p*-value
	*N* = 1750	*N* = 3,444	
Age	50.62 ± 7.43	51.36 ± 7.58	0.001
Woman(%)	882 (50.4%)	1,642 (47.7%)	0.034
Married	1,672 (95.5%)	3,298 (95.8%)	0.382
Smoking	604 (34.6%)	1,250 (36.4)	0.196
Drinking	69 (3.9%)	186 (5.4%)	0.012
Education			0.192
Uneducated	257 (14.7%)	539 (15.7%)	
Educated	1,493 (85.3%)	2,905 (84.3%)	
Income	2.0 (1.0–3.0)	2.0 (1.0–3.0)	<0.001
Farmers	1,519 (86.8%)	3,238 (91.1%)	<0.001
BMI			0.432
<23 kg/m^2^	655 (37.4%)	1,293 (37.6%)	
23- < 25 kg/m^2^	502 (28.7%)	995 (28.9%)	
≥25 kg/m^2^	593 (33.9%)	1,155 (33.5%)	
*Helicobacter pylori* infection	771 (44.3%)	1863 (54.3%)	<0.001
Waist (Mean ± SD/cm)	85.57 ± 8.73	84.77 ± 8.97	0.002
Eating hot food	977 (55.8%)	2068 (60.1%)	0.002
Eating fast	350 (20.0%)	687 (19.9%)	0.496
TC	4.52 (0.0–12.7)	4.64 (0.0–9.2)	0.003
TG	1.50 (0.0–12.4)	1.51 (0.0–17.0)	0.464
HDL	1.13 (0.0–2.46)	1.13 (0.0–2.61)	0.723
LDL	2.75 (0.0–7.34)	2.86 (0.0–6.42)	0.000

Model 1 was adjusted for age, sex, marital status, education level, occupation, and household income. Model 2 was further adjusted for smoking status, alcohol consumption, dietary habits, body mass index, *Helicobacter pylori* infection, history of gastritis, history of peptic ulcer, history of hepatitis, history of gallbladder disease, history of polyps, hypertension.

### Serum lipid levels and atrophic gastritis risk

**Figure 2:**
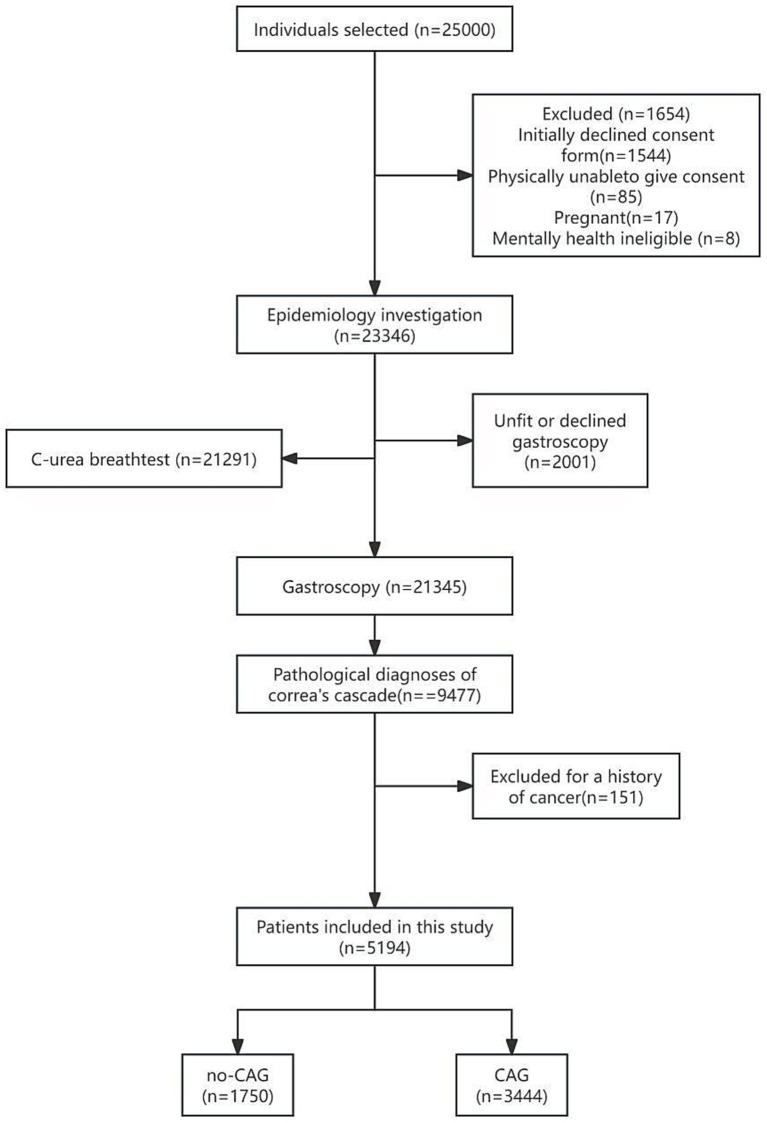
Restricted cubic spline analysis of the association between total cholesterol and prevalent chronic atrophic gastritis.

Table 2 displays the association between serum lipid levels and prevalent CAG after adjustment for multiple covariates. Logistic regression analyses, after controlling for intrinsic variables including age, sex, marital status, education, occupation, and household income, revealed that elevated total cholesterol posed a risk for developing chronic atrophic gastritis (CAG) compared to the no-CAG group (OR = 1.08, 95% CI = 1.01–1.15). Similarly, increased low-density lipoprotein levels were associated with a higher risk of atrophic gastritis (OR = 1.20, 95% CI = 1.10–1.32). Upon further adjustment for smoking, alcohol consumption, dietary habits (e.g., consumption of hot foods, fast eating), body mass index (BMI), *Helicobacter pylori* infection status, and medical history (e.g., gastritis, peptic ulcer, hepatitis, gallbladder disease, polyps, hypertension), there were no significant alterations in the outcomes. Subsequently, we categorized cholesterol, triglycerides, HDL, and LDL into quartiles and compared the characteristics among these four subgroups. Next, we explored the relationship between lipid levels and atrophic gastritis using a binary logistic regression model. Our findings indicate a significant positive trend between total cholesterol levels and the incidence of atrophic gastritis (refer to Table 2). Restricted cubic spline analysis of the association between total cholesterol and prevalent chronic atrophic gastritis(refer to Figure 2). The model was adjusted for age, sex, marital status, education, occupation, household income, smoking status, alcohol consumption, dietary habits, body mass index, *Helicobacter pylori* infection, and medical history. The solid line represents the estimated odds ratio, and the shaded area represents the 95% confidence interval. All available observations were included in the primary analysis. Sensitivity analyses using winsorized lipid values at the 1st and 99th percentiles showed similar results. Additionally, in the mutually adjusted model, high levels of LDL exhibited a similar elevation in the risk of atrophic gastritis compared to low levels of LDL (refer to Table 2). However, none of the models indicated a statistically significant association between HDL, triglycerides, and atrophic gastritis.

**Table 2:** The association between blood lipid and CAG.

Blood lipid	Model 1		Model 2	
	OR [95%CI]	*P* for trend	OR [95%CI]	*P* for trend
TC continues	1.07 (1.01–1.14)	0.030	1.10 (1.03–1.17)	0.010
TC (mmol/L, *median IQR*)				
Q1 (3.61 [<4])	Reference		Reference	
Q2 (4.32 [4–4.61])	1.24 (1.01–1.51)		1.26 (1.02–1.53)	
Q3 (4.93 [4.61–5.32])	1.35 (1.10–1.65)		1.40 (1.13–1.72)	
Q4 (5.90 [>5.32])	1.32 (1.08–1.62)	0.007	1.41 (1.14–1.73)	0.001
TG continues	1.05 (0.98–1.13)	0.170	1.07 (0.99–1.16)	0.060
TG (mmol/L, *median IQR*)				
Q1 (0.88 [<1.10])	Reference		Reference	
Q2 (1.29 [1.10–1.51])	1.04 (0.85–1.28)		1.08 (0.88–1.33)	
Q3 (1.75 [1.51–2.10])	1.04 (0.85–1.28)		1.09 (0.88–1.34)	
Q4 (2.71 [>2.10])	1.05 (0.86–1.29)	0.693	1.10 (0.89–1.36)	0.401
LDL continues	1.20 (1.10–1.32)	0.000	1.21 (1.10–1.34)	0.000
LDL (mmol/L, *median IQR*)				
Q1 (2.07 [<2.37])	Reference		Reference	
Q2 (2.61 [2.37–2.82])	1.19 (0.97–1.45)		1.20 (0.97–1.47)	
Q3 (3.06 [2.82–3.34])	1.24 (1.01–1.51)		1.26 (1.03–1.55)	
Q4 (3.73 [>3.34])	1.44 (1.17–1.76)	0.001	1.45 (1.18–1.79)	0.000
HDL continues	1.02 (0.79–1.31)	0.890	1.01 (0.78–1.30)	0.970
HDL (mmol/L, *median IQR*)				
Q1 (0.86 [<0.96])	Reference		Reference	
Q2 (1.06 [0.96–1.13])	1.01 (0.83–1.24)		1.03 (0.84–1.26)	
Q3 (1.23 [1.13–1.33])	1.04 (0.85–1.28)		1.04 (0.84–1.29)	
Q4 (1.49 [>1.33])	0.95 (0.77–1.62)	0.633	0.95 (0.76–1.17)	0.599

## Discussion

In this cross-sectional analysis of 5,194 participants from the Wuwei cohort, we observed that higher total cholesterol and LDL cholesterol levels were associated with greater odds of prevalent chronic atrophic gastritis. These associations remained after adjustment for sociodemographic factors, lifestyle factors, dietary habits, body mass index, *Helicobacter pylori* infection, and relevant medical history. In contrast, triglycerides and HDL cholesterol were not significantly associated with prevalent chronic atrophic gastritis. Because this study was cross-sectional, these findings should be interpreted as associations rather than evidence that elevated cholesterol or LDL cholesterol causes atrophic gastritis.

Chronic atrophic gastritis (CAG) represents a prevalent digestive disorder and is positively linked with the incidence of gastric cancer. Numerous domestic and international investigations have indicated that factors such as age, environmental conditions, genetic predisposition, dietary habits, *Helicobacter pylori* (Hp) infection, bile reflux, NSAID usage, and psychosocial factors contribute to CAG (4, 5). Our previous research has identified age, agricultural occupation, low annual household income, Hp infection, alcohol consumption, consumption of hot foods, and a history of gastritis or peptic ulcers as associated factors in CAG development (6). Recent studies have also suggested a potential role of lipid metabolism disorders in promoting atrophic gastritis progression (7). However, limited research has explored the impact of lipid metabolism on atrophic gastritis. In this study, we aimed to investigate the influence of lipid metabolism levels on atrophic gastritis, aiming to offer insights for the prevention and treatment of this condition and to aid in the management of gastrointestinal neoplastic diseases.

Previous studies examining the relationship between serum lipid levels and atrophic gastritis have reported inconsistent findings (8). Some studies suggested that higher triglyceride levels may be associated with atrophic gastritis, whereas HDL cholesterol may show an inverse association. Other studies did not observe clear associations for total cholesterol or LDL cholesterol. In contrast, our study found that higher total cholesterol and LDL cholesterol levels were associated with higher odds of prevalent chronic atrophic gastritis, while triglycerides and HDL cholesterol were not significantly associated. Differences in study populations, definitions of atrophic gastritis, lipid measurement methods, *Helicobacter pylori* status, and covariate adjustment may partly explain these inconsistent findings.

By analyzing the correlation between lipid levels and chronic atrophic gastritis (CAG), we observed no significant abnormalities in triglyceride (TG) and high-density lipoprotein cholesterol (HDL-C) levels between the non-atrophic gastritis and atrophic gastritis groups. However, TC and LDL-C levels were higher in the CAG group than in the No-CAG group. Because serum lipid levels and gastric mucosal status were assessed at the same time point, the direction of this association cannot be determined (4, 9, 10). Furthermore, older age was independently associated with prevalent CAG. Older individuals exhibited a higher susceptibility to atrophic gastritis, which aligns with previous studies indicating a higher prevalence of lipid metabolism disorders in older age groups. Thus, the association between atrophic gastritis and lipid metabolism disorders may be age-related. To address potential confounding factors such as age, sex, marital status, education, occupation, and household income, we conducted a binary logistic regression analysis. The results revealed a higher likelihood of atrophic gastritis in individuals with hypercholesterolemia (high total cholesterol levels). This finding is consistent with previous research by Kyoichi Adachi et al., who observed elevated serum total bile acids in subjects with greater gastric mucosal atrophy. Their study suggested that gastric mucosal atrophy may be related to changes in lipid metabolism after *H. pylori* eradication. However, in the present cross-sectional study, we could not determine the temporal direction of the association between lipid levels and CAG., with *H. pylori* clearance being particularly impactful in cases of more severe atrophy (11). Subsequent adjustments for additional risk factors, including smoking, alcohol consumption, dietary habits (e.g., consumption of hot food, eating speed), body mass index (BMI), *H. pylori* infection status, and medical history (e.g., gastritis, peptic ulcer, hepatitis, gallbladder disease, polyps, and hypertension), did not alter the previously observed results.

Several biological mechanisms may potentially explain the observed associations, although these mechanisms cannot be confirmed by the present cross-sectional study. First, dyslipidemia is often accompanied by chronic low-grade inflammation, which may be related to gastric mucosal inflammatory changes (12, 13). Second, elevated LDL cholesterol may be associated with oxidative stress, endothelial dysfunction, and impaired microvascular circulation, which could be linked to gastric mucosal vulnerability. Third, lipid abnormalities may coexist with unhealthy dietary patterns, smoking, alcohol consumption, and metabolic disorders, all of which may be associated with gastric mucosal lesions. Finally, *Helicobacter pylori* infection may interact with host metabolic status and systemic inflammatory responses. However, the directionality of these relationships remains uncertain. It is also possible that chronic gastric inflammation, dietary changes, or other unmeasured factors influence serum lipid levels (14–16).

The most important limitation of this study is its cross-sectional design. Serum lipid levels and gastric pathological status were measured at the same time; therefore, the temporal sequence between dyslipidemia and chronic atrophic gastritis cannot be determined. The observed associations do not establish that higher total cholesterol or LDL cholesterol preceded the occurrence of atrophic gastritis. Reverse causation is also possible, as chronic gastric mucosal disease may influence dietary intake, metabolism, or systemic inflammatory status, which in turn may affect lipid levels. Therefore, the findings should not be interpreted as evidence of causality. Prospective cohort studies with repeated lipid measurements and longitudinal assessment of gastric mucosal changes are needed to clarify temporal and causal relationships.

Despite these limitations, this study has several strengths. The analysis was based on a relatively large population from a high-risk region for gastric cancer, and all participants underwent gastroscopy with pathological assessment, which reduced outcome misclassification compared with studies relying only on symptoms or serological markers. In addition, the study considered a broad range of demographic, lifestyle, dietary, clinical, and *H. pylori*-related variables, allowing a more comprehensive assessment of the association between serum lipid profiles and prevalent CAG.

## Conclusion

In this cross-sectional study from the Wuwei cohort, higher total cholesterol and LDL cholesterol levels were associated with greater odds of prevalent chronic atrophic gastritis, whereas triglycerides and HDL cholesterol showed no significant association. These findings suggest a relationship between lipid metabolism and atrophic gastritis, but the cross-sectional design precludes conclusions regarding temporality or causality. Further prospective studies are warranted to determine whether lipid abnormalities precede the development or progression of atrophic gastritis and to clarify the underlying biological mechanisms.

## Data Availability

The original contributions presented in the study are included in the article/supplementary material, further inquiries can be directed to the corresponding authors.
